# Correlates of Pro-Drinking Practices in Drinking Parents of Adolescents in Hong Kong

**DOI:** 10.1371/journal.pone.0119554

**Published:** 2015-03-18

**Authors:** Wing Man Au, Sai Yin Ho, Man Ping Wang, Wing Sze Lo, Sze Pui Pamela Tin, Rong Huang, Tai Hing Lam

**Affiliations:** 1 School of Public Health, The University of Hong Kong, Hong Kong; 2 School of Nursing, The University of Hong Kong, Hong Kong; Cardiff University, UNITED KINGDOM

## Abstract

**Introduction and Aims:**

Parental alcohol-related practices are important risk factors of adolescent drinking, but little is known about the factors associated with these parental pro-drinking practices (PPDPs). We investigated the correlates of 9 PPDPs in drinking parents of adolescents in Hong Kong.

**Methods:**

A total of 2200 students (age 14.8±2.0; boys 63.2%) participated in a school-based cross-sectional survey in 2012. Analysis was restricted to 1087 (61.8%) students with at least 1 drinking parent as PPDPs were much more common in these families. Logistic regression was used to identify correlates of each PPDP.

**Results:**

Among 1087 students, the prevalence of PPDPs ranged from 8.2% for training drinking capacity to 65.7% for seeing parents drink. Only 14.8% of students had not experienced any of these practices. More frequent maternal drinking predicted parental training of drinking capacity. Older age predicted helping parents buy alcohol and parental encouragement of drinking. Adolescent girls were more likely to have received parental training of drinking capacity than boys. Higher perceived family affluence was associated with hearing parents saying benefits of drinking, and helping parents open bottle and pour alcohol.

**Conclusions:**

PPDPs were associated with parental drinking frequency and various socio-demographic factors. These results have implications on alcohol control programmes involving parents to tailor messages for reducing PPDPs based on the characteristics of adolescents and parents.

## Introduction

Parental drinking is a well-known risk factor of adolescent drinking [1–4]. However, various alcohol-related parental practices may also directly or indirectly promote drinking in adolescents. For example, parental provision of alcohol and home alcohol availability were associated with adolescent drinking [5–7], and alcohol availability at home was associated with the number of alcohol-related problems experienced by adolescents [3]. A recent study also found that fetching or pouring alcohol for adults was associated with adolescent alcohol sipping [7].

We have recently identified 9 parental practices that may potentially promote alcohol drinking in Hong Kong Chinese adolescents [8]. These parental pro-drinking practices (PPDPs) include seeing parents drink and drunk; hearing parents mention the benefits of drinking and certain alcohol taste good; helping parents buy alcohol, open bottle and pour alcohol; and encouragement of drinking and training of drinking capacity (the ability to drink more without getting drunk). Exposure to these PPDPs was associated with alcohol drinking in adolescents, the results of which will be reported separately.

Studying adolescent and parental factors associated with these practices may help identify adolescents at risk of such exposures and understand reasons behind these practices. However, such studies are scarce and all based on Western populations. In an American study, mothers who drank heavily were more likely to be younger, born outside the United States, and had higher education [9]. An European study has found alcohol availability at home associated with higher family socioeconomic status [3]. However, an Australian study found no significant association between parental supply of alcohol and socio-demographic characteristics, including sex and birth order of child; and sex, household income, place of birth and religion of parents [10].

Despite alcohol use is glamorized with its purported health [11] and psychological benefits [12], the drinking prevalence in Hong Kong remains relatively low. The adolescent drinking prevalence (past 30 days) was 19% in Hong Kong Chinese adolescents, which was much lower than that of 39% in the United States [13, 14]. Adolescent drinking is generally regarded as inappropriate [15, 16] and parental influence tends to be strong in Chinese families [17, 18], therefore, PPDPs may have a relatively large effect on adolescent drinking in these families.

In the present study, we investigated the correlates of PPDPs in Hong Kong Chinese adolescents. We hypothesized that these practices are generally associated with parental drinking frequency and age of adolescents. Moderate alcohol drinking has been associated with higher socioeconomic status, suggesting that this group is more health conscious and may drink for the perceived benefits of alcohol [19]. Similarly, middle class parents have been reported to encourage drinking in children [20]. We, therefore, also hypothesized that positive comments about alcohol and direct encouragement of drinking are associated with higher socioeconomic status of parents.

## Methods

### Participants and procedure

A total of 2200 Secondary 1 (Grade 7 in the United States) to 6 students (age 14.8±2.0, boys 63.2%) from 4 randomly selected schools, including 2 co-education and 2 boy schools in different districts of Hong Kong were recruited for a school-based cross-sectional study in 2012. In order to recruit 4 schools, invitations to 8 schools were made with a response rate of 50%. Time and administrative issues were the main reasons for school refusals. Response rate at the student level was 92% and non-participation was due to absence from school. Parents and students were informed of the survey through an invitation letter. No reply was required for participation and declining parents were to ask their children to return a blank questionnaire during the survey. Student participation was voluntary even with parental consent. Students completed an anonymous questionnaire independently in classrooms. Teachers were present to maintain classroom order and provide guidance based on survey instructions. Completed questionnaires were immediately collected and sealed in an opaque envelope in front of students. Ethical approval was granted by the Institutional Review Board of the University of Hong Kong/Hospital Authority Hong Kong West Cluster (UW 12–421), including consent procedure with written consent waived.

The students with missing data (mainly parental drinking status 16.8% and PPDPs 0.9%) were excluded. To account for the differences between our sample and the underlying population in Hong Kong (Cohen effect size 0.06 for age and 0.23 for sex), the remaining 1757 students were weighted to reflect the age and sex distributions of the corresponding general population based on census data for the calculation of prevalence estimates in the present study [21]. Weighting was applied using a weighting factor determined by the sex-age combination of each subject. For example, as girls aged 16 were under-represented, these subjects were given a weighting factor of greater than 1. As PPDPs were much more common in families with drinking parents, the present study was restricted to 1087 students (age 14.6±1.6; boys 50.2%) with at least one drinking parent.

### Measurements

#### PPDPs

As a comprehensive list of such practices was not available in the literature, these 9 PPDPs were based on observations, discussions with adolescents and parents, and anecdotal reports. Students were asked “Have you experienced the following situations?” with response options categorized into 4 groups: 1. Saw parents a) drink and b) being drunk; 2. Heard parents mention a) benefits of drinking and b) alcohol tasted good; 3. Helped parents a) buy alcohol, b) open bottle and c) pour alcohol; and 4. Parental actions a) encouraged me to drink and b) trained my drinking capacity. Students chose each option that was applicable. These pro-drinking practices in each student were analysed as individual practices and the total number of practices (0, 1–2, 3–4, 5 or above).

#### Parental drinking

Paternal and maternal drinking were assessed by the items “How often did your father/mother drink alcohol in the past 30 days?” in two separate items each with 5 options: “never”, “seldom”, “sometimes”, “always”, “unknown”.

#### Socio-demographic factors

Age, sex, family structure, place of birth, highest parental education and perceived family affluence were included as socio-demographic factors. Age was dichotomised as junior (11 or below to 14) and senior (15 or above); family structure as intact (parents together) and non-intact (divorced/live separately, mother died, father died, both died or others); and place of birth as Hong Kong and outside Hong Kong (China, Macau, Taiwan or others). Highest parental education was classified as primary or below, secondary, and tertiary. Perceived family affluence was categorized into low (relatively poor, below average), average, and high (above average, relatively rich).

### Statistical analysis

Adjusted odds ratios (AORs) and 95% confidence interval (95% CI) were computed using STATA 10.1. Logistic regression yielded AORs and 95% CI for each PPDP by the above-mentioned socio-demographic factors adjusting for each other and school clustering. School clustering was adjusted using the command “robust clust (school variable)” in STATA 10.1. Linear regression was used to derive the regression coefficients for the number of PPDPs with similar adjustments.

## Results

Table 1 shows that after weighting, among 1087 students with one or both drinking parents, half (50.2%) were boys, 63.2% were aged 15 or below, 80.9% were with an intact family and 74.8% were born in Hong Kong. Half the students (49.7%) had parents with secondary education and 59.7% reported average family affluence. Parental drinking frequency was most commonly reported to be seldom (paternal 42.8%, maternal 40.1%), followed by sometimes (paternal 27.9%, maternal 15.3%) and always (paternal 17.9%, maternal 3.9%). The prevalence of PPDPs ranged from 8.2% for training drinking capacity to 65.7% for seeing parents drink. Only 14.8% of students had not experienced any of these practices.

**Table 1:** Characteristics of subjects with at least one current drinking parent.

Variables	Characteristics	N (%)	Weighted N (%)^a^
Age	Junior (≤15)	680 (63.3)	687 (63.2)
	Senior (≥16)	394 (36.7)	400 (36.8)
Sex	Male	716 (66.7)	546 (50.2)
	Female	358 (33.3)	541 (49.8)
Family structure	Intact	873 (81.6)	877 (80.9)
	Non-intact	197 (18.4)	207 (19.1)
Place of birth	Hong Kong	802 (75.4)	805 (74.8)
	Others	262 (24.6)	272 (25.2)
Highest parental education	Primary or below	311 (29.0)	290 (26.6)
	Secondary	539 (50.2)	540 (49.7)
	Tertiary	224 (20.9)	258 (23.7)
Perceived family affluence	Low	267 (24.9)	258 (24.2)
	Medium	619 (57.6)	636 (59.7)
	High	166 (15.5)	172 (16.1)
Paternal drinking	None	68 (6.4)	85 (7.9)
	Seldom	446 (41.5)	465 (42.8)
	Sometimes	311 (29.0)	303 (27.9)
	Always	212 (19.7)	194 (17.9)
	Unknown	37 (3.4)	39 (3.6)
Maternal drinking	None	439 (40.9)	418 (38.5)
	Seldom	429 (39.9)	435 (40.1)
	Sometimes	137 (12.8)	167 (15.3)
	Always	44 (4.1)	43 (3.9)
	unknown	25 (2.3)	24 (2.2)
Parental pro-drinking practices	**Parents were seen**		
	Drinking	705 (65.6)	714 (65.7)
	Drunk	312 (29.1)	317 (29.1)
	**Parents were heard saying**		
	Benefits of drinking	128 (11.9)	138 (12.7)
	Alcohol tasted good	347 (32.3)	382 (35.1)
	**Parents being helped**		
	Buy alcohol	285 (26.5)	287 (26.4)
	Open bottle	346 (32.2)	331 (30.4)
	Pour alcohol	356 (33.1)	376 (34.6)
	**Parental actions**		
	Encouraged drinking	128 (11.9)	148 (13.6)
	Trained drinking capacity	77 (7.2)	89 (8.2)
Number of parental pro-drinking practices	0	172 (16.0)	161 (14.8)
	1–2	439 (40.9)	448 (41.2)
	3–4	273 (25.4)	273 (25.2)
	5 or above	190 (17.7)	204 (18.8)

^a^ Weighted by age and sex of the corresponding population in Hong Kong

Table 2 shows that seeing parents drink was negatively associated with being senior students (AOR 0.70, 95% CI 0.52–0.94). The AOR (95% CI) of seeing parents drink increased with paternal drinking frequency (seldom: 1.94, 1.10–3.42; sometimes: 2.10, 1.18–3.74; always: 5.06, 2.67–9.58; P for trend<0.001) but decreased with maternal drinking frequency (seldom: 1.65, 1.21–2.25; sometimes: 1.54, 0.98–2.43; always: 0.94, 0.46–1.91; P for trend<0.001).

**Table 2:** Prevalence and adjusted odds ratios (AORs) for parents being seen drinking and drunk.

Characteristics	Level	Parents were seen drinking			Parents were seen drunk		
		Prevalence	AOR^a^	95% CI	Prevalence	AOR^a^	95% CI
Age	≤15	68.5	1		28.6	1	
	≥16	61.0	0.70*	0.52, 0.94	30.0	1.05	0.77, 1.44
Sex	Male	63.9	1		30.2	1	
	Female	67.5	1.21	0.89, 1.65	28.1	1.02	0.73, 1.41
Place of birth	Hong Kong	64.9	1		28.7	1	
	Others	67.4	1.23	0.90, 1.68	31.4	1.03	0.74, 1.43
Family structure	Intact	67.7	1		27.7	1	
	Non-intact	57.0	0.71	0.49, 1.03	35.1	1.25	0.85, 1.85
Perceived family affluence	Low	56.0	1		37.8	1	
	Medium	72.1	0.94	0.67, 1.30	33.4	0.84	0.60, 1.19
	High	64.9	0.81	0.52, 1.26	25.9	0.91	0.57, 1.45
	P for trend = 0.225				P for trend = 0.333		
Highest parental education	Primary	61.2	1		29.3	1	
	Secondary	68.6	1.30	0.95, 1.76	30.4	1.13	0.81, 1.57
	Tertiary	64.7	1.22	0.82, 1.80	26.4	0.77	0.49, 1.20
	P for trend = 0.117				P for trend = 0.707		
Paternal drinking	None	45.5	1		27.2	1	
	Seldom	64.2	1.94*	1.10, 3.42	19.6	0.63	0.34, 1.17
	Sometimes	65.9	2.10*	1.18, 3.74	29.0	1.11	0.60, 2.06
	Always	79.6	5.06***	2.67, 9.58	52.4	2.87**	1.52, 5.41
	Unknown	58.6	2.08	0.85, 5.04	34.2	1.04	0.42, 2.59
	P for trend (excluding unknown) = <0.001				P for trend (excluding unknown) = <0.001		
Maternal drinking	None	64.3	1		23.0	1	
	Seldom	67.9	1.65**	1.21, 2.25	26.6	1.31	0.94, 1.84
	Sometimes	67.0	1.54	0.98, 2.43	46.4	2.21*	1.40, 3.47
	Always	64.3	0.94	0.46, 1.91	49.8	2.02**	1.01, 4.05
	Unknown	43.2	0.73	0.30, 1.74	25.8	0.94	0.36, 2.46
	P for trend (excluding unknown) = 0.202				P for trend (excluding unknown) = <0.001		

^a^Adjusted odds ratios were mutually adjusted and were adjusted for school clustering effects

*p<0.05,

**p<0.01,

***p<0.001

Table 3 shows that hearing the benefits of alcohol was associated with high perceived family affluence (AOR: 1.97, 95% CI: 1.09–3.56; P for trend = 0.035) and maternal drinking (seldom: 1.89, 1.81–3.01; always: 4.59, 1.91–11.00; P for trend<0.001). Hearing alcohol tasted good was associated with female students (1.64, 1.21–2.22), tertiary parental education (1.95, 1.30–2.92; P for trend = 0.002) and maternal drinking (seldom: 2.02, 1.46–2.78; sometimes: 2.44, 1.57–3.80; P for trend<0.001).

**Table 3:** Prevalence and adjusted odds ratios (AORs) for parents being heard saying the benefits of drinking and alcohol tasted good.

Characteristics	Level	Benefits of drinking			Alcohol tasted good		
		Prevalence	AOR^a^	95% CI	Prevalence	AOR^a^	95% CI
Age	≤15	12.8	1		35.8	1	
	≥16	12.7	1.27	0.82, 1.98	34.0	1.08	0.80, 1.47
Sex	Male	11.5	1		28.2	1	
	Female	14.0	0.96	0.63, 1.47	42.1	1.64**	1.21, 2.22
Place of birth	Hong Kong	13.4	1		36.1	1	
	Others	10.3	0.83	0.51, 1.35	31.4	0.91	0.65, 1.25
Family structure	Intact	13.9	1		35.5	1	
	Non-intact	7.8	0.56	0.29, 1.06	33.5	0.86	0.58, 1.27
Perceived family affluence	Low	10.2	1		28.8	1	
	Medium	11.5	0.93	0.56, 1.55	34.2	1.05	0.74, 1.48
	High	20.6	1.97*	1.09, 3.56	47.3	1.45	0.93, 2.27
	P for trend = 0.035			P for trend = 0.089			
Highest parental education	Primary	8.7	1		26.7	1	
	Secondary	10.8	1.38	0.84, 2.29	32.7	1.25	0.90, 1.75
	Tertiary	21.3	1.69	0.95, 2.98	49.8	1.95**	1.30, 2.92
	P for trend = 0.030			P for trend = 0.002			
Paternal drinking	None	14.1	1		52.3	1	
	Seldom	13.1	0.78	0.37, 1.68	28.8	0.70	0.39, 1.24
	Sometimes	13.2	0.98	0.45, 2.11	38.0	1.08	0.60, 1.93
	Always	11.5	0.55	0.24, 1.30	39.9	1.16	0.63, 2.13
	Unknown	5.9	0.47	0.09, 2.44	29.8	0.68	0.27, 1.76
	P for trend (excluding unknown) = 0.337			P for trend (excluding unknown) = 0.067			
Maternal drinking	None	8.2	1		24.6	1	
	Seldom	14.1	1.89**	1.18, 3.01	36.7	2.02***	1.46, 2.78
	Sometimes	17.8	1.81	0.94, 3.48	57.2	2.44***	1.57, 3.80
	Always	24.7	4.59**	1.91, 11.00	45.6	1.85	0.91, 3.74
	Unknown	10.0	1.74	0.37, 8.10	19.0	0.98	0.34, 2.80
	P for trend (excluding unknown) = <0.001			P for trend (excluding unknown) = <0.001			

^a^Adjusted odds ratios were mutually adjusted and were adjusted for school clustering effects

*p<0.05,

**p<0.01,

***p<0.001

Table 4 shows that helping parents buy alcohol was associated positively with being senior students (AOR: 1.67, 95% CI: 1.21–2.31), born outside Hong Kong (2.18, 1.57–3.02) and paternal drinking (sometimes: 4.01, 1.76–9.14, always: 6.55, 2.83–15.17, P for trend<0.001), and negatively with tertiary parental education (0.58, 0.36–0.93; P for trend = 0.023). Helping parents open bottle was associated with high perceived family affluence (1.58, 1.01–2.48; P for trend = 0.045), paternal (always: 3.44, 1.81–6.53; P for trend<0.001) and maternal drinking (seldom: 2.11, 1.53–2.91; sometimes: 2.69, 1.72–4.19; always: 3.42, 1.70–6.89; P for trend<0.001). Helping parents pour alcohol was associated with female students (1.44, 1.06–1.96), high perceived family affluence (1.61, 1.03–2.52; P for trend = 0.065), paternal drinking (seldom: 2.13, 1.09–4.15; sometimes: 3.29, 1.67–6.46; always: 4.63, 2.30–9.29; P for trend<0.001) and maternal drinking (seldom: 2.10, 1.54–2.89; sometimes: 2.36, 1.52–3.67; always: 3.35, 1.68–6.68; P for trend< 0.001).

**Table 4:** Prevalence and adjusted odds ratios (AORs) for parents being helped to buy, open bottle and pour alcohol.

Characteristics	Level	Buy alcohol			Open bottle			Pour alcohol		
		Prevalence	AOR^a^	95% CI	Prevalence	AOR^a^	95% CI	Prevalence	AOR^a^	95% CI
Age	≤15	22.0	1		28.9	1		33.0	1	
	≥16	34.1	1.67**	1.21, 2.31	33.1	1.16	0.86, 1.58	37.5	1.22	0.90, 1.65
Sex	Male	27.9	1		33.3	1		30.3	1	
	Female	24.9	1.13	0.80, 1.61	27.5	0.86	0.62, 1.18	39.0	1.44*	1.06, 1.96
Place of birth	Hong Kong	22.2	1		30.6	1		33.7	1	
	Others	38.8	2.18***	1.57, 3.02	30.5	1.08	0.78, 1.48	36.5	1.14	0.83, 1.56
Family structure	Intact	24.4	1		30.4	1		35.0	1	
	Non-intact	35.4	1.19	0.79, 1.77	31.0	1.02	0.69, 1.49	33.7	0.86	0.58, 1.28
Perceived family affluence	Low	32.6	1		27.7	1		32.9	1	
	Medium	24.6	0.87	0.61, 1.24	27.2	1.15	0.82, 1.63	32.3	1.06	0.75, 1.49
	High	23.8	0.84	0.51, 1.38	44.0	1.58*	1.01, 2.48	44.3	1.61*	1.03, 2.52
	P for trend = 0.45				P for trend = 0.045			P for trend = 0.065		
Highest parental education	Primary	30.0	1		30.0	1		33.2	1	
	Secondary	28.1	0.83	0.60, 1.17	30.6	1.08	0.78, 1.49	33.0	1.12	0.81, 1.54
	Tertiary	18.8	0.58*	0.36, 0.93	30.5	1.14	0.76, 1.73	39.5	1.30	0.86, 1.95
	P for trend = 0.023				P for trend = 0.457			P for trend = 0.167		
Paternal drinking	None	11.8	1	21.3	1			17.9	1	
	Seldom	19.4	1.92	0.84, 4.38	22.8	1.21	0.66, 2.24	29.8	2.13*	1.09, 4.15
	Sometimes	28.6	4.01**	1.76, 9.14	31.8	1.75	0.94, 3.24	38.4	3.29**	1.67, 6.46
	Always	45.6	6.55***	2.83, 15.17	52.0	3.44***	1.81, 6.53	46.6	4.63***	2.30, 9.29
	Unknown	27.5	2.37	0.79, 7.10	21.3	0.99	0.38, 2.58	35.4	2.54	0.96, 6.69
	P for trend (excluding unknown) = <0.001				P for trend (excluding unknown) = <0.001			P for trend (excluding unknown) = <0.001		
Maternal drinking	None	22.7	1		21.8	1		24.5	1	
	Seldom	24.3	1.41	1.00, 2.00	34.1	2.11***	1.53, 2.91	38.9	2.10***	1.54, 2.89
	Sometimes	35.2	1.47	0.91, 2.40	37.9	2.69***	1.72, 4.19	45.3	2.36***	1.52, 3.67
	Always	39.1	1.88	0.91, 3.89	55.6	3.42**	1.70, 6.89	59.1	3.35**	1.68, 6.68
	Unknown	45.3	1.49	0.60, 3.71	15.6	0.55	0.18, 1.71	15.4	0.60	0.19, 1.85
	P for trend (excluding unknown) = 0.045				P for trend (excluding unknown) = <0.001			P for trend (excluding unknown) = <0.001		

^a^Adjusted odds ratios were mutually adjusted and were adjusted for school clustering effects

*p<0.05,

**p<0.01,

***p<0.001

Table 5 shows that parental encouragement of drinking was associated positively with being senior students (AOR: 1.60, 95% CI: 1.03–2.48), and negatively with being born outside Hong Kong (0.59, 0.36–0.98) and paternal drinking (seldom: 0.45, 0.21–0.95; P for trend = 0.208). Parental training of drinking capacity was associated with female students (2.28, 1.31–3.99) and maternal drinking (seldom: 2.18, 1.11–4.28; sometimes: 5.31, 2.52–11.19; always: 5.14, 1.81–14.57; P for trend<0.001).

**Table 5:** Prevalence and adjusted odds ratios (AORs) for parental encouragement of drinking and training of drinking capacity.

Characteristics	Level	Parental encouragement of drinking			Parental training of drinking capacity		
		Prevalence	AOR^a^	95% CI	Prevalence	AOR^a^	95% CI
Age	≤15	12.7	1		8.7	1	
	≥16	15.0	1.60*	1.03, 2.48	7.3	0.77	0.43, 1.36
Sex	Male	12.4	1		5.9	1	
	Female	14.8	1.03	0.66, 1.61	10.5	2.28**	1.31, 3.99
Place of birth	Hong Kong	14.7	1		8.8	1	
	Others	9.9	0.59*	0.36, 0.98	5.9	0.58	0.30, 1.11
Family structure	Intact	12.9	1		7.3	1	
	Non-intact	16.4	1.33	0.78, 2.24	11.9	1.79	0.97, 3.32
Perceived family affluence	Low	13.7	1		7.3	1	
	Medium	12.1	0.70	0.44, 1.12	6.3	0.95	0.50, 1.82
	High	17.5	1.09	0.60, 1.97	15.8	1.62	0.77, 3.42
	P for trend = 0.840				P for trend = 0.172		
Highest parental education	Primary	10.2	1		7.2	1	
	Secondary	13.4	1.30	0.80, 2.11	7.1	0.89	0.46, 1.59
	Tertiary	17.7	1.25	0.69, 2.26	11.6	1.28	0.64, 2.54
	P for trend = 0.392				P for trend = 0.358		
Paternal drinking	None	35.7	1		10.5	1	
	Seldom	8.3	0.45*	0.21, 0.95	7.7	1.14	0.43, 3.04
	Sometimes	13.0	0.72	0.34, 1.52	6.7	1.01	0.37, 2.73
	Always	17.6	0.75	0.34, 1.65	11.4	1.53	0.55, 4.26
	Unknown	12.7	0.28	0.07, 1.17	5.4	0.37	0.06, 2.13
	P for trend (excluding unknown) = 0.208				P for trend (excluding unknown) = 0.493		
Maternal drinking	None	8.8	1		3.9	1	
	Seldom	12.6	1.16	0.72, 1.86	8.6	2.18*	1.11, 4.28
	Sometimes	24.7	1.62	0.89, 2.96	16.1	5.31***	2.52, 11.19
	Always	30.5	2.06	0.87, 4.89	19.5	5.14**	1.81, 14.57
	Unknown	7.2	0.37	0.05, 2.89	0	-	-
	P for trend (excluding unknown) = 0.010				P for trend = <0.001		

^a^Adjusted odds ratios were mutually adjusted and were adjusted for school clustering effects

*p<0.05,

**p<0.01

***p<0.001

Table 6 shows that the number of parental pro-drinking practices was associated with being female students (regression coefficient 0.26, 95% CI 0.00–0.52), paternal (sometimes: 0.73, 0.21–1.24; always: 1.46, 0.92–1.99) and maternal drinking (seldom: 0.75, 0.50–1.02; sometimes: 1.13, 0.75–1.51; always: 1.29, 0.69–1.90).

**Table 6:** Mean (SD) and regression coefficient on the number of parental pro-drinking practices.

Characteristics	Level	Number of parental pro-drinking practices		
		Mean (SD)	Regression coefficient^a^	95% CI
Age	≤15	2.51 (1.98)	Reference	
	≥16	2.65 (2.03)	0.16	-0.09, 0.42
Sex	Male	2.44 (2.07)	Reference	
	Female	2.68 (1.91)	0.26*	0.00, 0.52
Place of birth	Hong Kong	2.53 (2.03)	Reference	
	Others	2.62 (1.89)	0.13	-0.13, 0.39
Family structure	Intact	2.55 (1.98)	Reference	
	Non-intact	2.62 (2.09)	-0.04	-0.36, 0.28
Perceived family affluence	Low	2.56 (1.84)	Reference	
	Medium	2.39 (1.92)	-0.07	-0.35, 0.21
	High	3.11 (2.21)	0.32	-0.05, 0.70
			P for trend = 0.074	
Highest parental education	Primary	2.36 (2.06)	Reference	
	Secondary	2.55 (1.90)	0.17	-0.10, 0.43
	Tertiary	2.80 (2.09)	0.22	-0.11, 56
			P for trend = 0.123	
Paternal drinking	None	2.36 (1.68)	Reference	
	Seldom	2.14 (1.87)	0.18	-0.32, 0.69
	Sometimes	2.65 (2.03)	0.73**	0.21, 1.24
	Always	3.57 (2.06)	1.46***	0.92, 1.99
	Unknown	2.31 (1.57)	0.12	-0.67, 0.90
			P for trend <0.001 (excluding unknown)	
Maternal drinking	None	2.02 (1.77)	Reference	
	Seldom	2.64 (1.93)	0.76***	0.50, 1.02
	Sometimes	3.48 (2.05)	1.13***	0.75, 1.51
	Always	3.88 (2.52)	1.29***	0.69, 1.90
	Unknown	1.81 (1.84)	-0.26	-1.03, 0.52
			P for trend <0.001 (excluding unknown)	

^a^Regression coefficients were adjusted for other factors shown in table and school clustering effects

## Discussion

We found that PPDPs were correlated with various socio-demographic factors. Older age predicted helping parents buy alcohol and parental encouragement of drinking. It is well known that adolescent drinking increases with age [22]. Our findings also suggest a concurrent increase in parental acceptance of adolescent drinking. The law in Hong Kong does not restrict children from buying alcohol in retail stores and the legal drinking age of 18 is only applicable to licensed premises such as bars and restaurants. It is unlikely that parents would encourage heavy drinking, but parents who support drinking during adolescence or early adulthood may promote drinking to prepare for their future drinking occasions [23].

Adolescent girls were more likely to have received parental training in drinking capacity. Female drunkenness is often linked to sexual harassment and unprotected sex [24, 25]. Parents might have trained drinking capacity in adolescent girls to protect them from these harms, although evidence for its effectiveness is lacking. Adolescent girls were also more likely to have heard their parents saying alcohol tasted good. Parents might have used taste to encourage girls to drink, although it is also possible that girls were more concerned about taste and hence could recall better than boys. Pouring alcohol for parents was also more commonly reported by girls, reflecting their greater involvement in house chores than boys [26, 27].

Supporting the hypothesis that pro-drinking practices are associated with higher socioeconomic status, higher perceived family affluence predicted hearing parents saying benefits of drinking, and helping parents open bottle and pour alcohol. Moreover, tertiary parental education also predicted parents saying alcohol tasted good. Higher socioeconomic status has been linked to health consciousness [28]. Similarly, our results suggested that parents with higher socioeconomic status tended to drink for pleasure and health, as was reported among red wine drinkers [29]. As regards family structure, students from non-intact families were less likely to report seeing parents drink, which may reflect their fewer contact hours with parents [30].

Students born outside Hong Kong, mainly from Mainland China, were more likely to report helping parents buy alcohol. The type of alcohol bought was not recorded but beer or Chinese wines seemed more likely as they would be easier for adolescents to identify and buy than the vast variety of Western wines and spirits. This speculation is consistent with the beer drinking preference of people with lower socioeconomic status that is more common among new immigrants from the Mainland [31]. These parents were less likely to encourage adolescent drinking, suggesting that health and social status were not their main reasons for drinking.

The results of the present study would be useful for family-based and school-based alcohol control programmes involving parents to tailor contents targeting various PPDPs according to the socio-demographic characteristics of students (age, sex, place of birth) and parents (education level, socioeconomic status). Our results also provide support for persuading parents to drink less, not to drink in front of children and not to involve them in buying alcohol, opening bottles and pouring alcohol. The present study would be particularly relevant also to Mainland Chinese parents who share similar cultural backgrounds.

Our study has several limitations. All data were reported by students including PPDPs and parental drinking, although adolescent reports were found to correlate with parental drinking practices [32]. Moreover, for social desirability, parents may tend to under-report their drinking-related practices, but adolescents would have little motivation in doing so. Having information on alcohol types would have enhanced the interpretation of results although it would not alter the conclusions of the present study. Finally, the cross-sectional correlations identified do not imply causal effects.

## Conclusions

PPDPs were associated with parental drinking frequency and various socio-demographic factors. These results have implications on alcohol control programmes involving parents to tailor messages for reducing PPDPs based on the characteristics of adolescents and parents.
